# Supplementary material for: Structural Plasticity Is a Feature of Rheostat Positions in the Human Na+/Taurocholate Cotransporting Polypeptide (NTCP)
Source: Int J Mol Sci. 2022 Mar 16;23(6):3211. doi: 10.3390/ijms23063211 (PMC8954283; doi:10.3390/ijms23063211)
Supplement: Supplementary file 1 [file ijms-23-03211-s001.zip › ijms-1601123-supplementary.pdf]

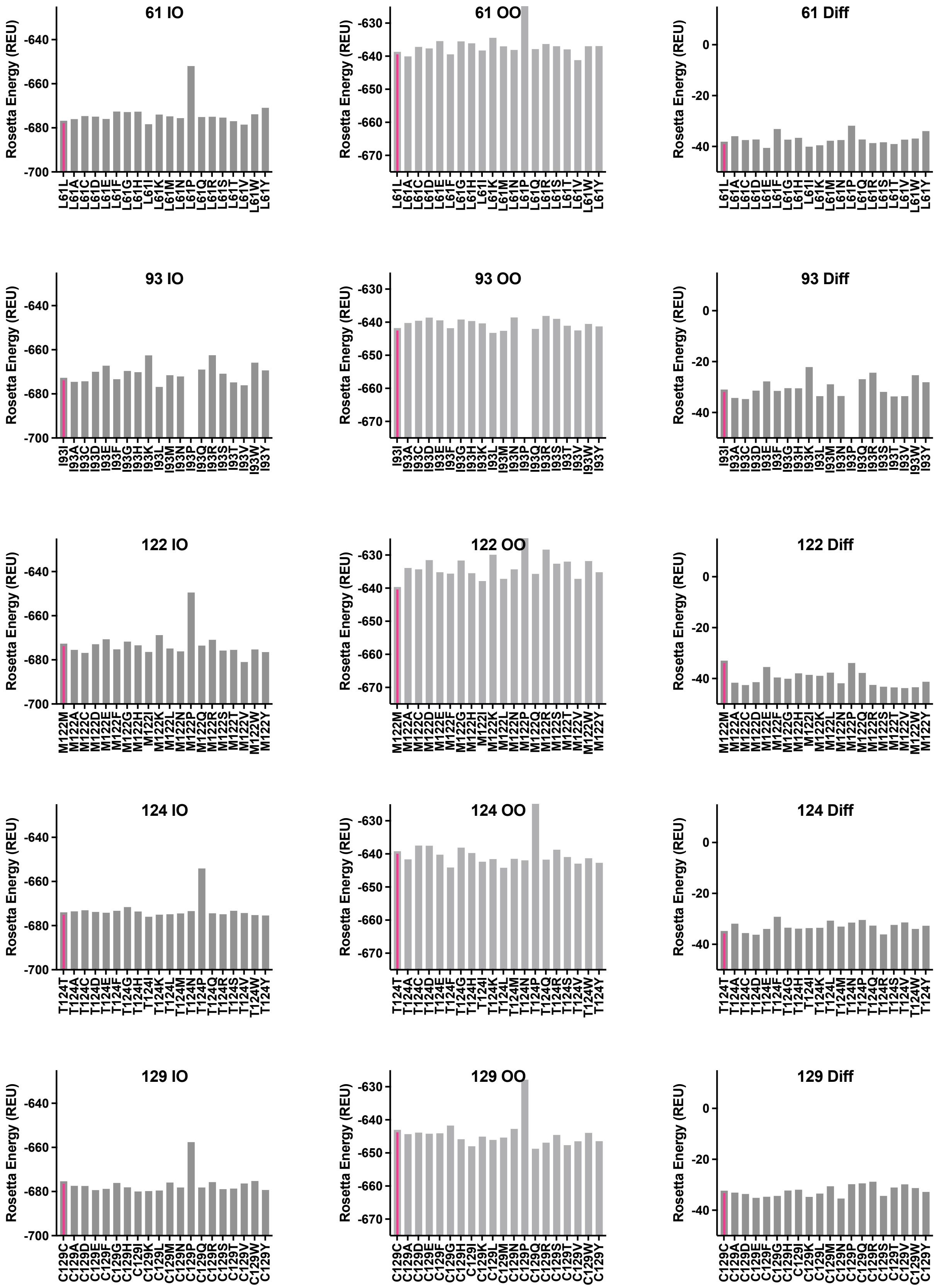

61 IO
61 OO
61 Diff
93 IO
93 OO
93 Diff
122 IO
122 OO
122 Diff
124 IO
124 OO
124 Diff
129 IO
129 OO
129 Diff
Rosetta Energy (REU)
-640 -660 -680 -700
-630 -640 -650 -660 -670
0 -20 -40
L61L L61A L61C L61D L61E L61F L61G L61H L61I L61K L61M L61N L61P L61Q L61R L61S L61T L61V L61W L61Y
I93I I93A I93C I93D I93E I93F I93G I93H I93K I93L I93M I93N I93P I93Q I93R I93S I93T I93V I93W I93Y
M122M M122A M122C M122D M122E M122F M122G M122H M122I M122K M122L M122N M122P M122Q M122R M122S M122T M122V M122W M122Y
T124T T124A T124C T124D T124E T124F T124G T124H T124I T124K T124L T124M T124N T124P T124Q T124R T124S T124V T124W T124Y
C129C C129A C129D C129E C129F C129G C129H C129I C129K C129L C129M C129N C129P C129Q C129R C129S C129T C129V C129W C129Y

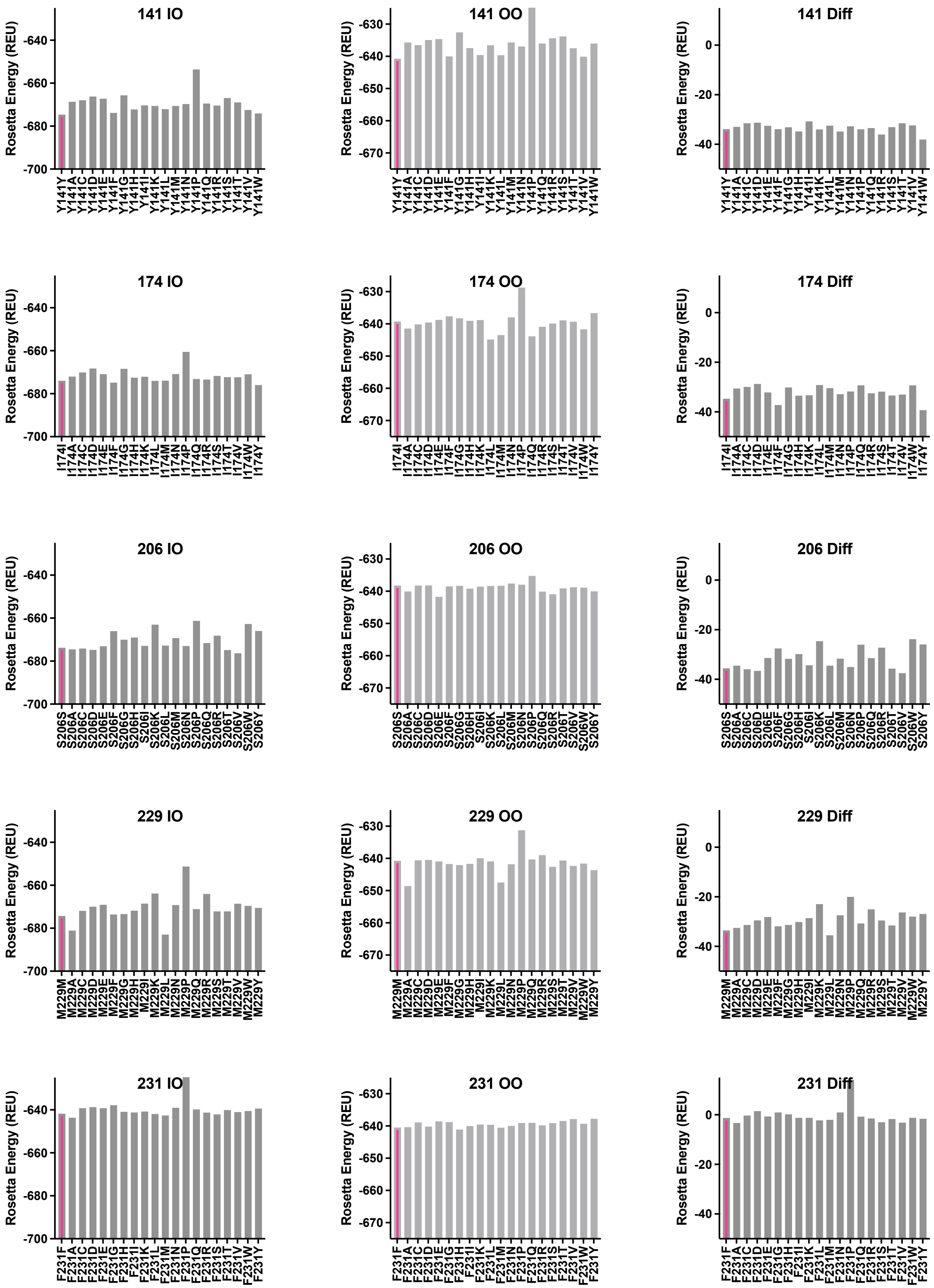
141 IO
141 OO
141 Diff
174 IO
174 OO
174 Diff
206 IO
206 OO
206 Diff
229 IO
229 OO
229 Diff
231 IO
231 OO
231 Diff
Rosetta Energy (REU)
Y141Y Y141A Y141C Y141D Y141E Y141F Y141G Y141H Y141I Y141K Y141L Y141M Y141N Y141P Y141Q Y141R Y141S Y141T Y141V Y141W
I174I I174A I174C I174D I174E I174F I174G I174H I174K I174L I174M I174N I174P I174Q I174R I174S I174T I174V I174W I174Y
S206S S206A S206C S206D S206E S206F S206G S206H S206I S206K S206L S206M S206N S206P S206Q S206R S206T S206V S206W S206Y
M229M M229A M229C M229D M229E M229F M229G M229H M229I M229K M229L M229N M229P M229Q M229R M229S M229T M229V M229W M229Y
F231F F231A F231C F231D F231E F231G F231H F231I F231K F231L F231M F231N F231P F231Q F231R F231S F231T F231V F231W F231Y

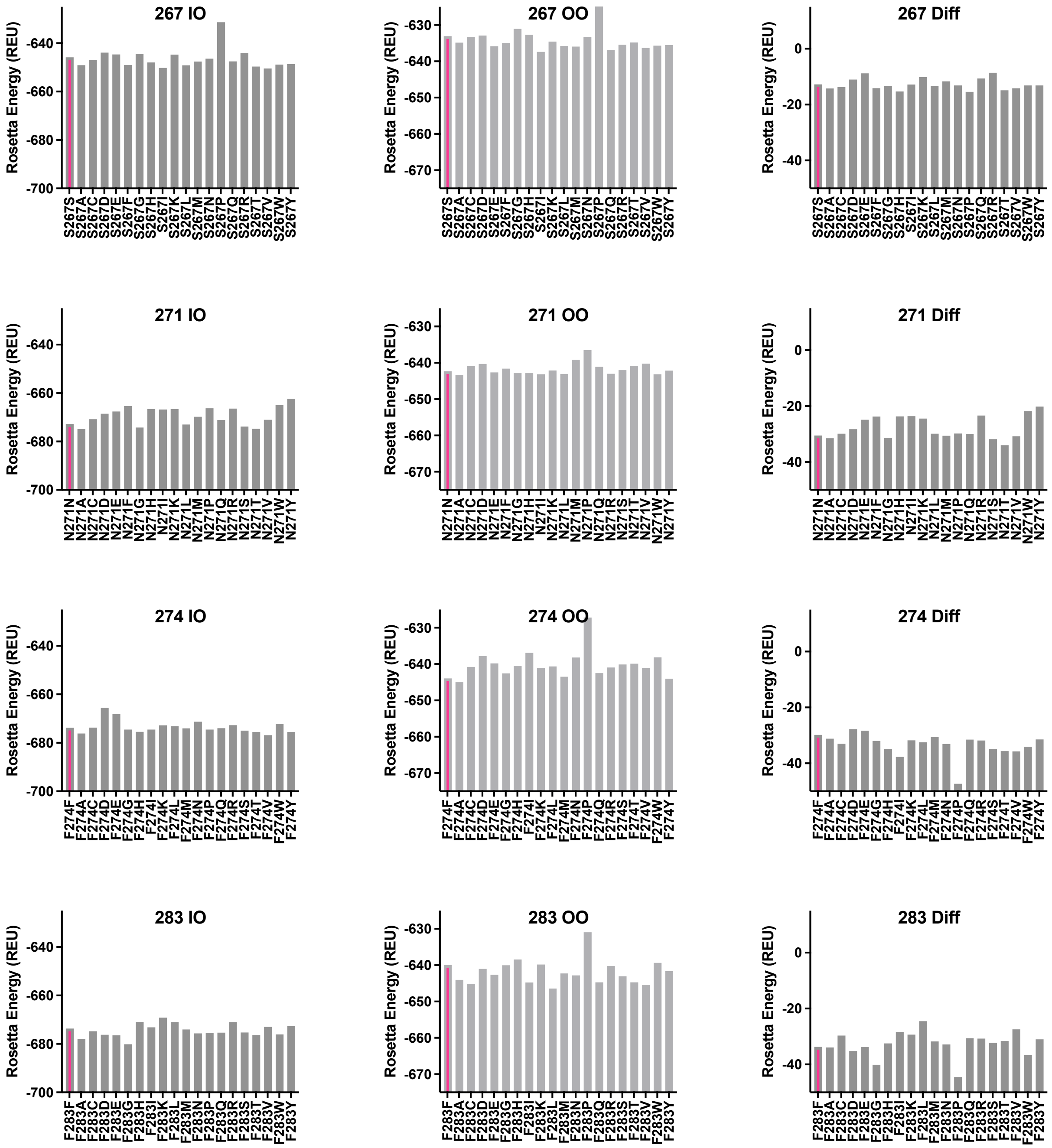

Figure S1. Rosetta energy scores for all 20 variants at all NTCP positions using the inward-open model (left column, “IO”), the outward-open model (middle column, “OO”), and the inward minus the outward-open model scores (right column, “Diff”). The magenta bar in each plot represents the wildtype control computation.

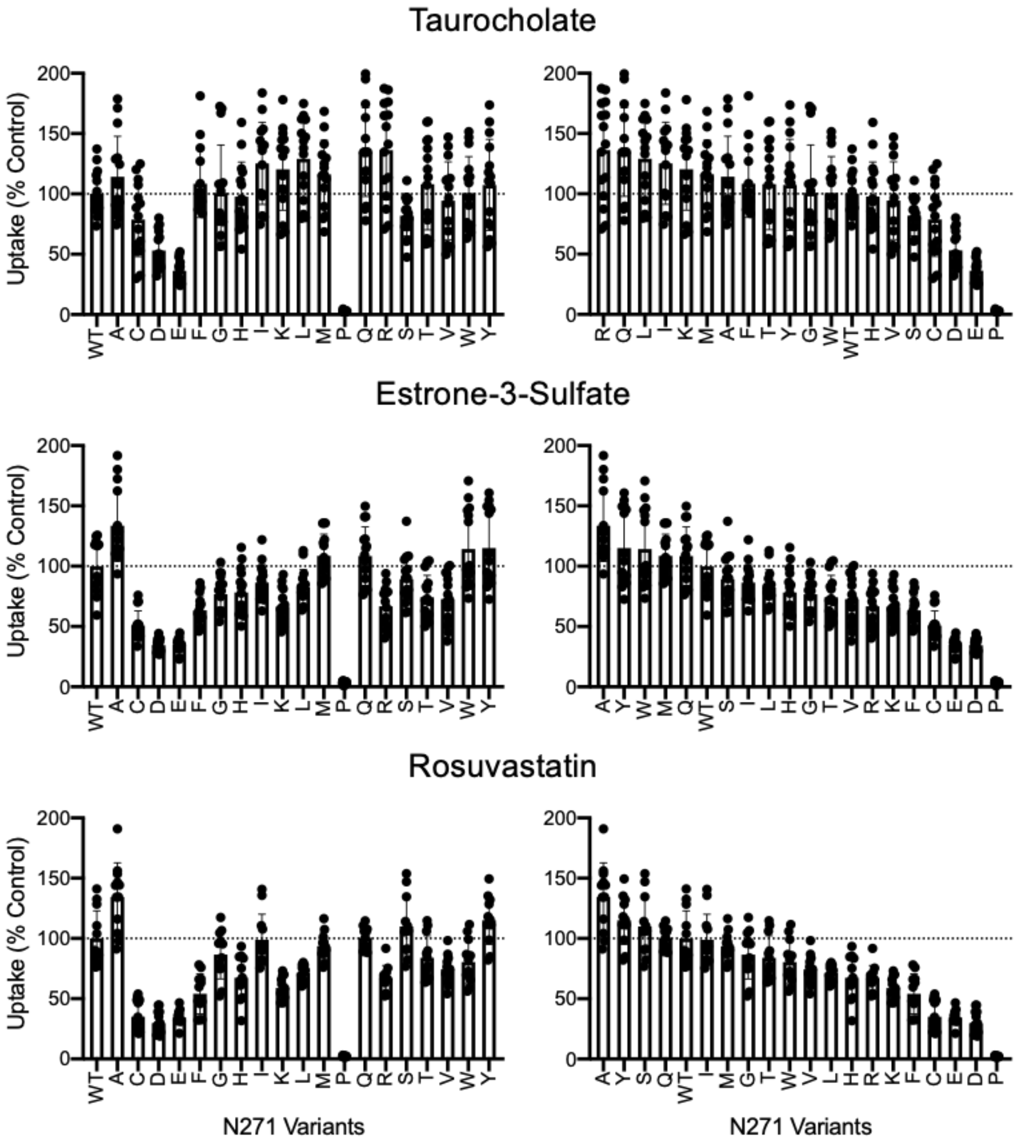

Figure S2: Transport of select substrates by wildtype NTCP and its 271 variants. HEK293 cells were transiently transfected with either wildtype NTCP (WT), N271 variants, or empty vector. Transport of ^{3}[H]taurocholate (30 nM), ^{3}[H]estrone-3-sulfate (5.8 nM) and ^{3}[H]rosuvastatin (50 nM) was measured at 37°C for 5 minutes, 48 hours post transfection. Uptake by the empty vector transfected cells was considered background and subtracted from the uptake by WT and N271 variant-expressing cells to yield net uptake. Further, results were calculated by setting wildtype to 100% and variants were calculated as a percent of wildtype NTCP. The left-hand side shows transport by the variants alphabetically by their amino acid substitutions, apart from wildtype which is listed first. The right-hand side shows the results ordered from highest to lowest transport activity. Individual data points from n=3 biological replicates with at least 2-3 technical replicates are reported with the bar indicating the mean of all replicates ± SD. Horizontal line indicated wildtype at 100% to aid in visual comparisons.

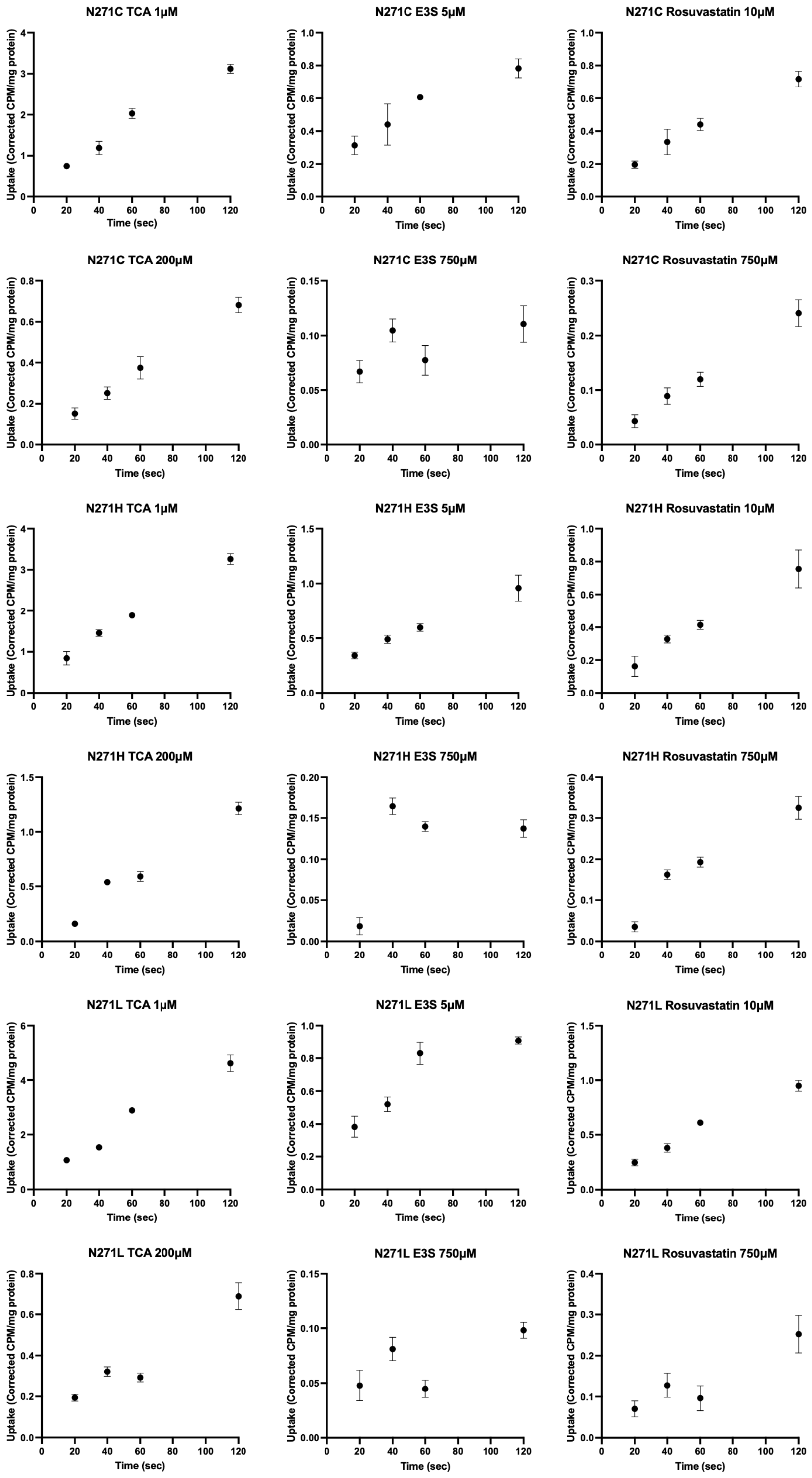

Figure S3: Time-dependent uptake of the different mutants at low and high substrate concentrations. Based on these results, kinetic experiments were performed for 30 seconds for taurocholate (TCA), 20 seconds for estrone-3-sulfate (E3S), and 30 seconds for rosuvastatin. A single experiment performed in triplicates is shown. Values were corrected for uptake into empty vector transfected cells and total protein. Means ± SD are shown.

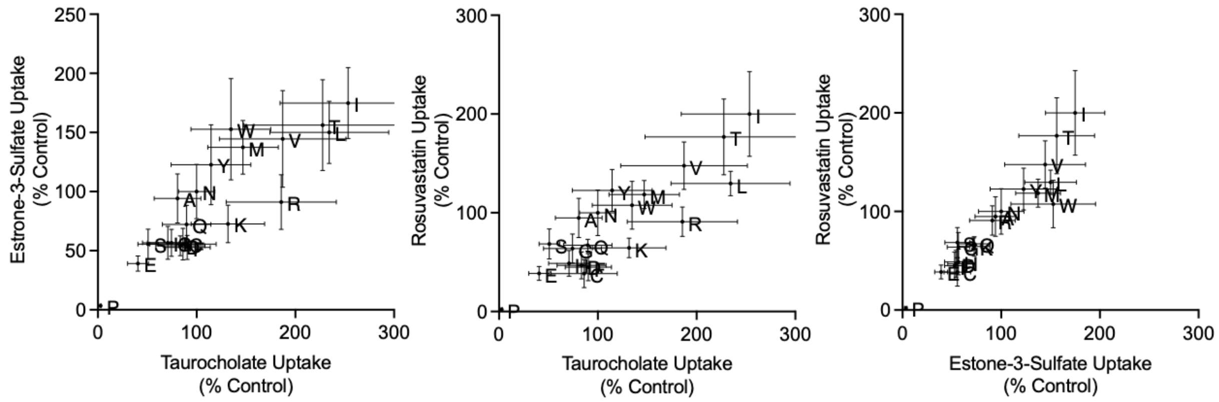

Figure S4: Correlation of normalized variant transport including standard deviations. The data of Figure 6 are plotted with standard deviations for both the X and Y axes.

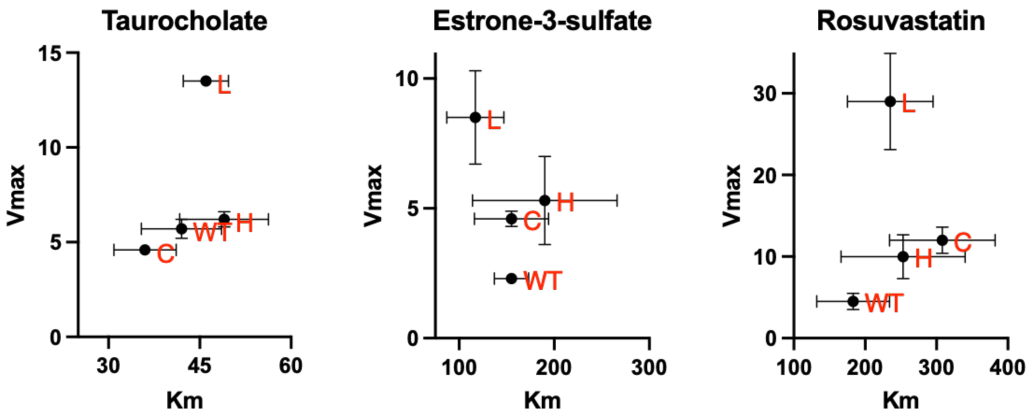

Figure S5: Correlation of Km and Vmax values for the uptake of the three substrates taurocholate, estrone-3-suflate and rosuvastatin. Values from Table 1 are plotted.

Table S1: N271 Pearson and Spearman Correlation Values

	**Pearson**		**Spearman**	
	Value	p-value	Value	p-value
Surface Expression v Rosetta Inward	-0.1782	0.4522	-0.09474	0.6912
Surface Expression v Rosetta Outward	-0.1075	0.6519	-0.05113	0.8305
Surface Expression v Inward Minus Outward	-0.1204	0.6130	-0.1609	0.4980
Taurocholate v Estrone-3-Sulfate	0.8802	**<0.0001**	0.8541	**<0.0001**
Taurocholate v Rosuvastatin	0.8807	**<0.0001**	0.8150	**<0.0001**
Estrone-3-Sulfate v Rosuvastatin	0.9527	**<0.0001**	0.9564	**<0.0001**
Taurocholate v Rosetta Inward	-0.08100	0.7342	0.02556	0.9148
Estrone-3-Sulfate v Rosetta Inward	-0.08745	0.7139	-0.1699	0.4738
Rosuvastatin v Rosetta Inward	-0.1733	0.4649	-0.2256	0.3390
Taurocholate v Rosetta Outward	-0.2838	0.2253	-0.1414	0.5522
Estrone-3-Sulfate v Rosetta Outward	-0.3026	0.1947	-0.3278	0.1582
Rosuvastatin v Rosetta Outward	-0.2894	0.2158	-0.2526	0.2826
Taurocholate v Rosetta Inward Minus Outward	0.04426	0.8530	0.1248	0.6001
Estrone-3-Sulfate v Rosetta Inward Minus Outward	0.04620	0.8466	-0.04211	0.8601
Rosuvastatin v Rosetta inward Minus Outward	-0.03923	0.8696	-0.1263	0.5957

Pearson (linear) and Spearman (rank order- nonparametric) correlation scores from Figure 4 and Figure 6 were calculated using GraphPad Prism 9. Significant correlations ($p>0.05$) are indicated in bold.
